# Differential Expression in the Tumor Microenvironment of mRNAs Closely Associated with Colorectal Cancer Metastasis

**DOI:** 10.1245/s10434-022-12574-1

**Published:** 2022-10-12

**Authors:** Kazuhiro Ito, Mitsumasa Osakabe, Ryo Sugimoto, Shun Yamada, Ayaka Sato, Noriyuki Uesugi, Naoki Yanagawa, Hiromu Suzuki, Tamotsu Sugai

**Affiliations:** 1 Department of Molecular Diagnostic Pathology, School of Medicine, Iwate Medical University, Shiwagun’yahabachou, Japan; 2 Department of Molecular Biology, Sapporo Medical University, Sapporo, Japan

## Abstract

**Background:**

Metastasis of colorectal cancer (CRC) is a major cause of CRC-related mortality. However, the detailed molecular mechanism of CRC metastasis remains unknown. A recent study showed that the tumor microenvironment, which includes cancer cells and the surrounding stromal cells, plays a major role in tumor invasion and metastasis. Identification of altered messenger RNA (mRNA) expression in the tumor microenvironment is essential to elucidation of the mechanisms responsible for tumor progression. This study investigated the mRNA expression of genes closely associated with metastatic CRC compared with non-metastatic CRC.

**Methods:**

The samples examined were divided into cancer tissue and isolated cancer stromal tissue. The study examined altered mRNA expression in the cancer tissues using The Cancer Genome Atlas (TCGA) (377cases) and in 17 stromal tissues obtained from our laboratory via stromal isolation using an array-based analysis. In addition, 259 patients with CRC were enrolled to identify the association of the candidate markers identified with the prognosis of patients with stage 2 or 3 CRC. The study examined the enriched pathways identified by gene set enrichment analysis (GSEA) based on the Kyoto Encyclopedia of Genes and Genomes (KEGG) module in both the TCGA dataset and isolated stromal tissue.

**Results:**

As a result, whereas tenascin-C, secreted phosphoprotein 1 and laminin were expressed in metastatic CRC cells, olfactory receptors (ORs) 11H1 and OR11H4 were expressed in stromal tissue cells isolated from metastatic CRC cases. Finally, upregulated expression of tenascin-C and OR11H4 was correlated with the outcome for CRC patients.

**Conclusion:**

The authors suggest that upregulated expression levels of tenascin-C and OR11H1 play an important role in CRC progression.

**Supplementary Information:**

The online version contains supplementary material available at 10.1245/s10434-022-12574-1.

Colorectal cancer (CRC) is a leading cause of cancer mortality in developed countries, especially Western nations and Japan. The World Health Organization (WHO) estimates that 945,000 new cases and 492,000 deaths occur yearly.^1^ Although the 5-year overall survival rate may improve with recent advances in therapies, the prognosis for patients with metastatic CRC remains poor.^2–4^ Metastasis is responsible for the majority of CRC deaths, mainly due to the high level of molecular alterations associated with metastatic CRC.^2–7^

Colorectal cancer is a highly heterogeneous disease that comprises different tumor phenotypes characterized by diverse molecular and morphologic alterations.^8,9^ Such heterogeneous changes may promote neoplastic progression and cancer metastasis.^8,9^ As a result, tumor clones expressing genes responsible for cancer progression and metastatic potential are selected as driver clones.^7–9^ Although several mechanisms associated with cancer progression and metastasis in CRC have been suggested,^3–5,7–9^ altered messenger RNA (mRNA) expression is recognized as the ultimate driver.^10–12^

It is very important to identify the gene transcripts that contribute to the invasive and metastatic potential of cancer cells.^10–12^ Despite the many proteins involved in cancer spread, some of these proteins are difficult to observe in patient tissue samples.^10–12^ Recently, advances in high-throughput analyses of mRNA expression, such as array-based analyses and bioinformatics, have been applied to evaluate tumor progression and metastatic spread in many cancers, including colorectal, gastric, ovarian, and lung cancers.^13–19^ Clinical and pathologic insights obtained from comprehensive analyses are critical for exploring biomarkers of disease progression and metastasis for anticancer therapies, as well as for guiding precise clinical decision-making for patients with progressive tumors.^15,18,19^

Recent studies have shown that the tumor microenvironment, including invasive cancer nests and the surrounding stromal cells, plays an essential role in tumor development and metastasis.^20–22^ Specific cancer-associated fibroblasts (CAFs) are thought to be major players in the formation of the microenvironment.^20–22^ According to this theory, not only cancer cells but also the surrounding stromal cells (especially CAFs) drive cancer progression, eventually leading to metastasis.^20–22^ Identification of the molecular alterations occurring in both cancer and stromal cells could help elucidate the molecular mechanisms of metastasis.

This study aimed to evaluate the predictive value of specific mRNAs from a metastasis-specific signature in an independent, clinically well-defined, prospectively collected sample of primary CRC cells and their surrounding stromal cells. For this purpose, samples of CRC with lymph node metastasis were analyzed to identify the genes with altered mRNA expression versus CRC without lymph node metastasis using an array-based analysis. Separate datasets, comprising The Cancer Genome Atlas (TCGA) data to evaluate CRC cells and our laboratory data to evaluate the surrounding cancer stromal cells, were used to explore altered mRNA expression. In addition, the immunohistochemical expression of the target markers identified in this report were examined to determine their associations with the prognosis of patients with stage 2 or 3 CRC.

## Materials and Methods

**Fig. 1:**
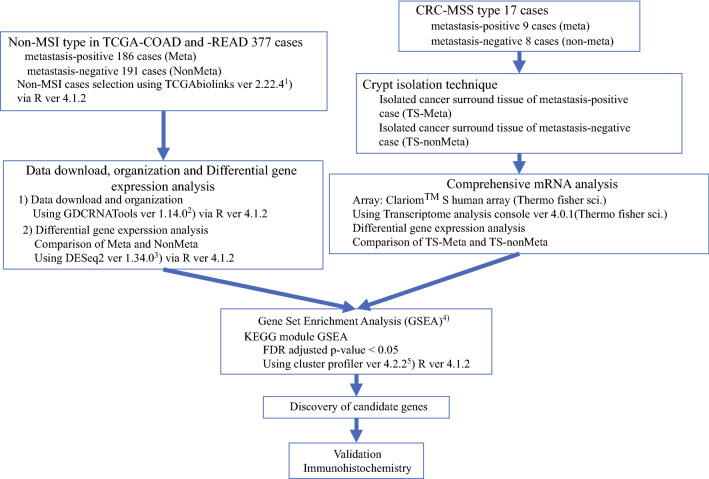
Work flow of this study

The work flow for this study is shown in Fig. 1.

### Clinicopathologic Characteristics of CRC Cases Obtained from TCGA (First Cohort)

The Cancer Genome Atlas (https://portal.gdc.cancer.gov/)^15^ is a publicly available, widely used gene expression database that contains many different types of samples and can be used in conjunction with gene analysis applications.^15^ Most importantly for our purposes, TCGA includes very detailed clinicopathologic information such as the presence of lymph node or distant metastasis and patient outcomes.^15^

We selected clinical data relevant to CRC with an MSS phenotype using TCGAbiolinks v2.22.41 via R v4.1.2.^23^ Next, expression data were downloaded and organized using GDCRNATools v1.14.0 via R v4.1.2.^24^ In addition, differential gene expression in metastatic CRC relative to non-metastatic CRC was analyzed using DESeq2 v1.34.0 via R v4.1.2.^25^ The clinical information (age, sex, and tumor stage) associated with 377 tumor tissue samples also was obtained. Detailed clinicopathologic data are provided in Table 1.

**Table 1:** Clinicopathologic findings of colorectal carcinoma

	TCGA dataset			Isolated cancer stroma			*P *value
	Total	Metastatic cases *n* (%)	Non-metastatic cases *n* (%)	Total	Metastatic cases *n* (%)	Non-metastatic cases *n* (%)	
Total	377	186 (49.3)	191 (50.7)	17	9 (52.9)	8 (47.1)	
Sex							N.S
Male	195 (51.7)	92 (49.5)	103 (53.9)	11 (64.7)	5 (55.6)	6 (75)	
Female	182 (48.3)	94 (50.5)	88 (46.1)	6 (35.3)	4 (44.4)	2 (25)	
Median age: years (range)	66 (31–90)	65 (31–90)	68 (35–90)	66 (43–81)	66 (43–77)	65.5 (56–81)	N.S
Location							N.S
C/A/T/D/S/R/unknown	62/39/41/15/104/105/11	36/15/15/9/53/52/6	26/24/26/6/51/53/5	2/2/1/3/6/3/0	2/2/1/2/2/0/0	0/0/0/1/4/3/0	
Median size: mm (range)				40 (15–90)	40 (15–60)	50 (22–90)	
Histologic type							
Moderately differentiated				17 (100)	9 (100)	8 (100)	
Stage							NS
1	63 (16.7)	0 (0)	63 (33)	1 (5.9)	0 (0)	1 (12.5)	
2	128 (34)	0 (0)	128 (67)	7 (41.2)	0 (0)	7 (87.5)	
3	127 (33.7)	127 (68.3)	0 (0)	7 (41.2)	7 (77.8)	0 (0)	
4	59 (15.6)	59 (31.7)	0 (0)	2 (11.8)	2 (22.2)	0 (0)	

C, cecum; A, ascending colon; T, transverse colon; D, descending colon; S, sigmoid colon; R, rectum; NS; not significant

### Clinicopathologic Characteristics of the CRC Cases from Which Stromal Tissue was Isolated (Second Cohort)

A total of 17 patients with CRC resected at Iwate Medical University between 2019 and 2021 were enrolled to evaluate the molecular alterations in the stromal tissue surrounding the cancer. Histologic examination was performed using hematoxylin and eosin staining of the tissues, and classification was performed according to the General Rules for Management of the Japanese Colorectal Cancer Association.^26^ The pathologic stage was determined in accordance with the World Health Organization (WHO).^27^ The clinicopathologic variables evaluated were age, sex, tumor location, stage and T stage, histologic type, and lymphatic/venous invasion (Table 1). Finally, patients who underwent preoperative chemoradiotherapy or emergency surgery were excluded.

This study was approved by the local ethics committee of Iwate Medical University (approval no. MH2018-042), and all the patients provided informed consent according to institutional guidelines.

### Colonic Gland and Stromal Isolation

Colonic crypt isolation from tumor and normal mucosal tissues to obtain pure gland and stromal tissues, respectively, was performed as described previously.^28,29^ Isolated tumor gland and stromal tissue samples were obtained from the central area of the tumor involving the invasive front. We confirmed inclusion of the invasive front by examining histologic sections. Cancer gland and stromal cells (mainly from fibrous tissue consisting of CAFs) were obtained separately under a dissecting microscope. In addition, normal gland and stromal tissues were collected as control tissues.

The stromal cells surrounding the cancer glands were carefully isolated from the tissues. The isolated glands and stromal cells were processed routinely into paraffin-embedded histologic sections to evaluate the histologic features. However, only the stromal cells were used for mRNA extraction given that isolated cancer glands were not examined for mRNA expression.

**Fig. 2:**
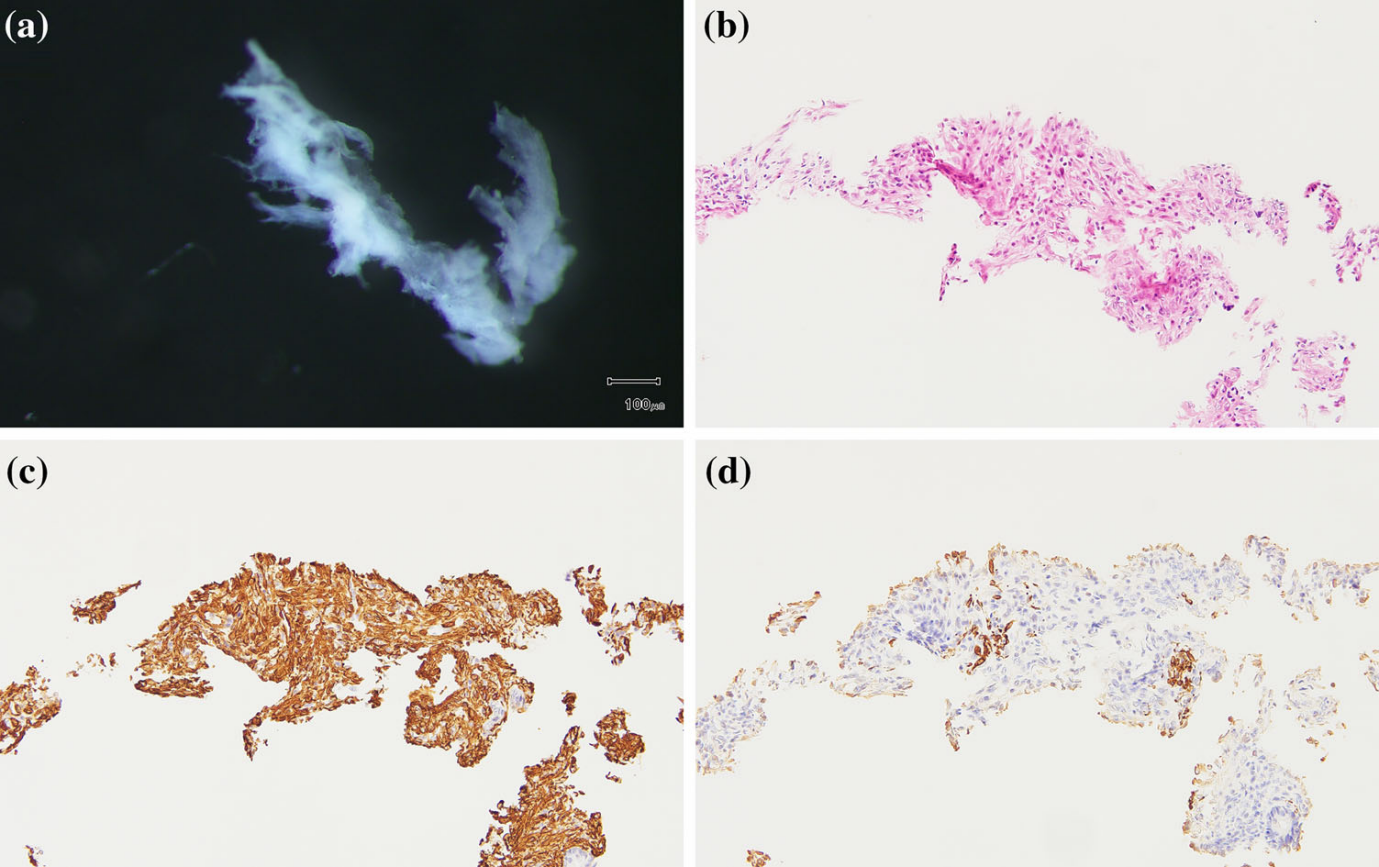
**a** Stromal cells as seen under a dissecting microscope. **b** Histology of cancer-associated fibroblasts in tissue sections stained with hematoxylin and eosin (HE). **c** Positive immunohistochemical expression of smooth muscle actin. **d** Negative immunohistochemical expression of desmin

The stromal cells were immunostained with antibodies targeting smooth muscle actin (clone 1A4; Dako, Carpinteria, CA, USA) and desmin (clone D33; Dako). We confirmed the exclusive presence of stromal cells according to negative smooth muscle actin and positive desmin immunostaining, which is indicative of smooth muscle cells. Contamination of other materials (e.g., cancer glands) was not evident in the gland or stromal tissue samples examined. However, we could not rule out the presence of other non-epithelial cells, such as inflammatory and vessel cells, among the stromal cells. Representative images are shown in Fig. 2.

### Clariom S Human Array and Gene Expression Analysis

For each array experiment, 500-ng total RNA (see the Supplementary Methods section for details regarding the RNA extraction method) was used for labeling before hybridization to the Clariom S human array (Thermo Fisher Scientific, Waltham, MA, USA). A total of 21,453 mRNAs are contained on this array. The probe labeling, chip hybridization, and scanning were performed according to the manufacturer’s instructions. The array data were generated using the Transcriptome Analysis Console (v4.0.1.36; Thermo Fisher Scientific), and differential gene expression was analyzed.

### GO and KEGG Enrichment Analyses

We performed gene set enrichment analysis (GSEA) based on KEGG pathway datasets using clusterProfiler v4.2.2 via R v4.1.2.^30,31^ We used an FDR-adjusted *p* value lower than 0.05 as the cut-off criterion for screening the enriched genes.

### Assessment of the Immunohistochemical Results

Shown are the immunostaining antibodies we used (Table S1). The study evaluated OR11H1 and OR11H4 immunohistochemical expression (see the Supplementary Methods section for details regarding the immunohistochemistry method) in the stromal fibroblast compartment of each tumor, whereas tenascin-C, laminin, and SPP1 expression was evaluated in the cancer cell component. Inflammatory cells were carefully excluded from the analysis. Only cytoplasmic expression of OR11H1, OR11H4, tenascin-C, laminin, and SPP1 was regarded as positive.

The immunostaining intensity and area were evaluated separately. The immunostaining intensity was classified into four categories and scored as follows: negative (0), weak (1), moderate (2), and strong (3). The immunostaining area for fusiform stromal cells was semi-quantified as follows: 0 % (score 0), 1 % to 25 % (score 1), 26 % to 50 % (score 2), and 51 % to 100 % (score 3). The sum of the immunostaining intensity and area scores was used as the final score (Table S2).^32^ A score greater than 3 was considered positive.^32^

**Fig. 3:**
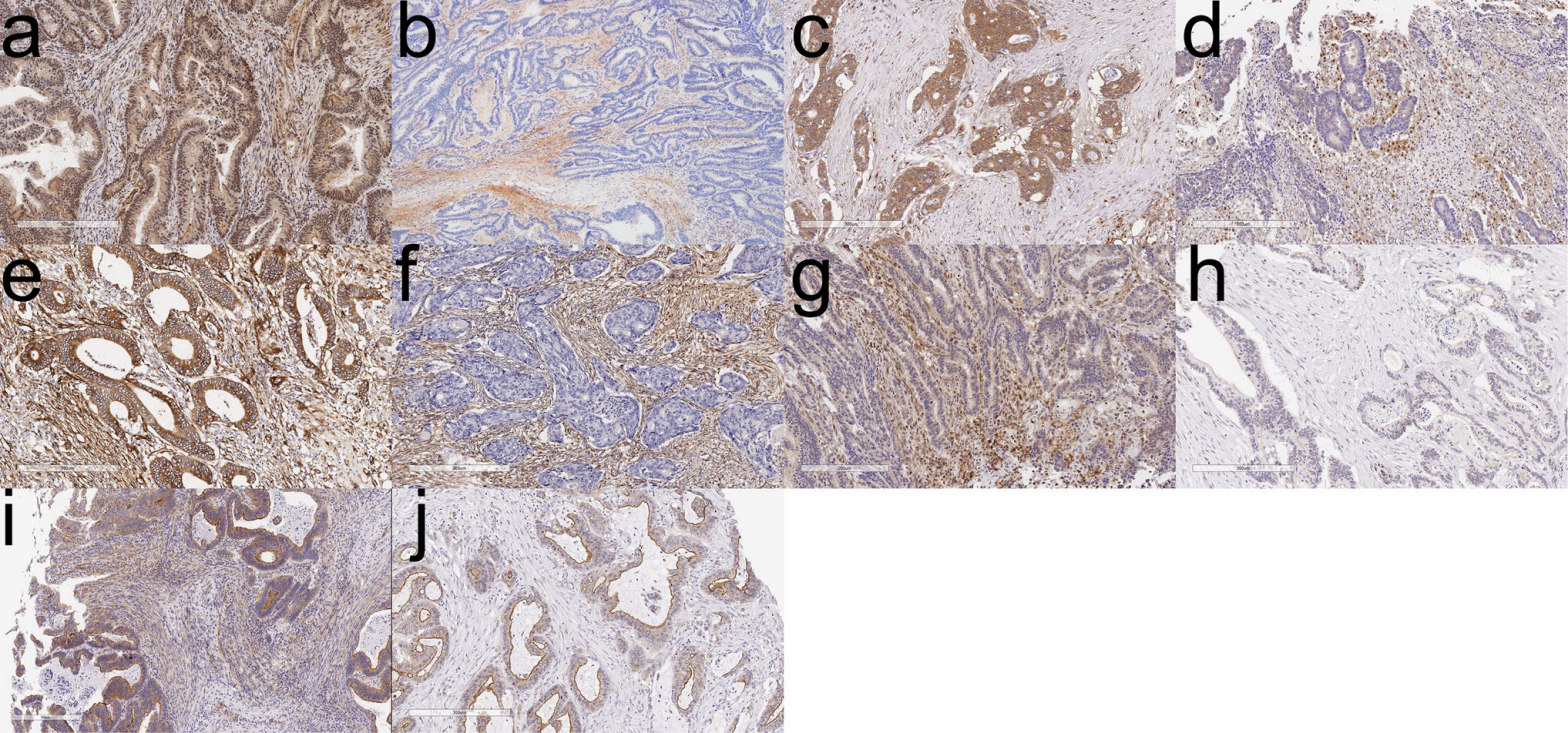
Representative histologic features. **a** Positive immunohistochemical expression of tenascin-C in metastatic colorectal cancer (CRC). **b** Negative immunohistochemical expression of tenascin-C in non-metastatic CRC. **c** Positive immunohistochemical expression of SPP1 in metastatic CRC. **d** Negative immunohistochemical expression of SPP1 in non-metastatic CRC. **e** Positive immunohistochemical expression of laminin in metastatic CRC. **f** Negative immunohistochemical expression of laminin in non-metastatic CRC. **g** Positive immunohistochemical expression of OP11H1 in metastatic CRC. **h** Negative immunohistochemical expression of OR11H1 in non-metastatic CRC. **i** Positive immunohistochemical expression of OP11H4 in metastatic CRC. **j** Negative immunohistochemical expression of OR11H4 in non-metastatic CRC

The scores were determined by expert diagnostic pathologists (M.O. and T.S.) blinded to the study end point. If the results among the pathologists were discordant, a discussion was held until a consensus was reached. Finally, representative histologic features are depicted in Fig. 3. “Immunohistochemistry,” “RNA and DNA extraction,” and “analysis of microsatellite instability (MSI)” are described in the Supplementary Methods section.

### Statistical Analysis

Data were analyzed using JMP Pro 16.2 software (SAS, Tokyo, Japan). The clinicopathologic variables (sex, location, pathologic T stage, and histologic type) were analyzed using Fisher’s exact test. Age distribution, overall survival, and disease-free survival were examined using the Mann–Whitney *U* test. Comparison of the immunohistochemical scores between the primary tumor site and the metastatic site was performed using the Wilcoxon signed-rank test.

Kaplan–Meier survival analyses were performed using the log-rank test for comparison of survival curves. Uni- and multivariate analyses were performed using Cox proportional hazards models to identify the variables that significantly predicted overall and disease-free survival. The level of significance was indicated by a *p* value lower than 0.05, and the confidence interval was determined at the 95 % level.

## Results

### Microsatellite Analysis of Isolated Cancer Glands

All tumors examined were classified into the microsatellite stable phenotype according to previously reported criteria.^33^

### Distribution of the Significantly Enriched Pathways Identified by KEGG Module GSEA in the TCGA Dataset and Stromal Tissue

**Fig. 4:**
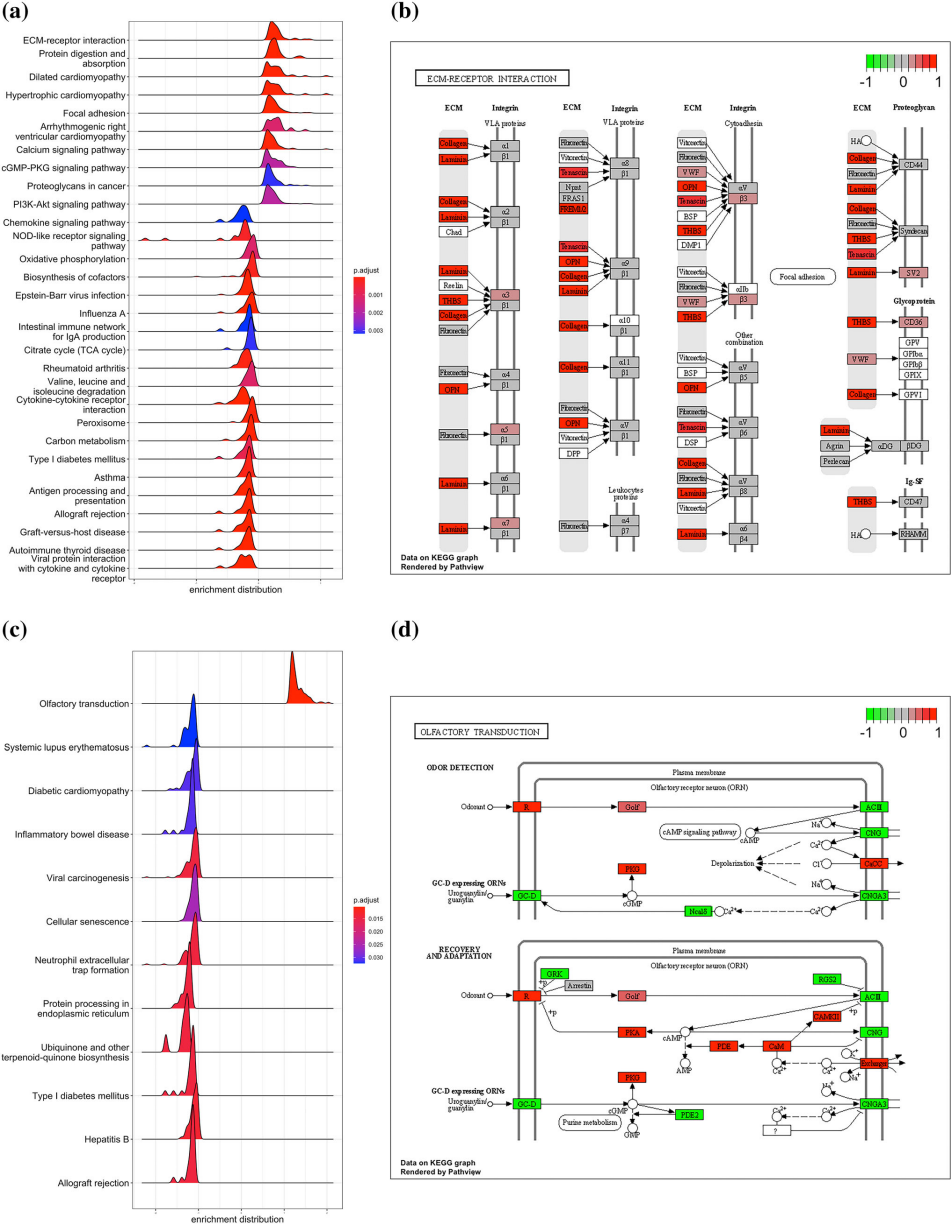
**a** A ridgeline plot showing the distribution of the most significantly enriched pathways identified by gene set enrichment analysis (GSEA) based on the Kyoto Encyclopedia of Genes and Genomes (KEGG) module in The Cancer Genome Atlas (TCGA) datasets. Benjamini–Hochberg/FDR-adjusted *p* values are displayed as a color gradient. **b** Visualization of the extracellular matrix (ECM)-receptor interaction pathway (hsa04512) as the most significantly enriched pathway. Gene expression values are displayed as a color gradient. **c** A ridgeline plot showing the distribution of the most significantly enriched pathways identified by KEGG module GSEA in the isolated cancer stroma. Benjamini–Hochberg/FDR-adjusted *p* values are displayed as a color gradient. **d** Visualization of the olfactory transduction pathway (hsa04740) as the most significantly enriched pathway. Gene expression values are displayed as a color gradient

We performed GSEA of the 377 CRC cases comprising 186 metastatic and 191 non-metastatic CRCs. The significantly enriched KEGG pathways identified by GSEA are shown in Table S3. The top 30 KEGG pathways are shown in Fig. 4a and b. The top 10 KEGG pathways were extracellular matrix (ECM) receptor interaction, protein digestion and absorption, dilated cardiomyopathy, hypertrophic cardiomegaly, focal adhesion, arrhythmogenic right ventricular cardiomyopathy, calcium-signaling pathway, cGMP-PKG-signaling pathway, proteoglycans in cancer, and PI3-Akt-signaling pathway. Of these, we focused on the ECM–receptor interaction pathway, which is closely associated with colorectal carcinogenesis. The highly expressed genes associated with this pathway and metastatic CRC are displayed in Fig. 4a and b and Table S4. Of these, tenascin-C, SPP1, and laminin were selected for further analysis because of their close association with CRC progression.

We performed GSEA of the 17 CAF samples isolated from nine metastatic and eight non-metastatic CRCs**.** The significantly enriched KEGG pathways identified by GSEA were olfactory transduction, hepatitis B, allograft rejection, neutrophil extracellular trap formation, protein-processing in endoplasmic reticulum, viral carcinogenesis, type 1 diabetes mellitus, ubiquinone and other terpenoid-quinone biosynthesis, cellular senescence, inflammatory bowel disease, diabetic cardiomyopathy, and systemic lupus erythematosus (Fig. 4c–d and Table S5).

We focused on the olfactory transduction pathway. The highly expressed genes associated with this pathway and metastatic CRC are shown in Fig. 4c and d and Table S6. Of these genes, OR11H1 and OR11H4 were selected for further analysis given that reliable and reproducible antibodies are commercially available for the proteins.

### Associations of the Clinicopathologic Findings Between Metastatic CRC and Non-metastatic CRC

We examined whether the candidate markers identified in the first and second cohorts were associated with the prognosis of the CRC patients. In addition, we compared the clinicopathologic variables listed in Table S7 between the patients with metastatic CRC and those with non-metastatic CRC (validation cohort). There were significant differences in the frequency of stage, overall survival, and disease-free survival according to the presence of metastasis. In addition, the median overall and disease-free survival values in this cohort were appropriate for analyzing the prognosis of patients with stage 2 or 3 CRC (Table S7).

### Survival Analyses in the First and Second Cohorts

Kaplan–Meier analyses were performed to determine the association between the disease-free survival frequencies and the cancer or stromal cells (Fig. S1). Although positive immunohistochemical expression of tenascin-C in cancer cells was associated with poorer overall survival (*p* < 0.0001), it was not associated with disease-free survival. There was a significant difference in the overall survival of patients with positive versus negative/weak immunohistochemical expression of OR11H4 in stromal cells.

### Uni- and Multivariate Analyses of the Associations of Clinicopathologic Variables and Cell Markers with Survival in Stages 2 and 3 CRC Patients

Univariate analyses using Cox proportional hazards models identified five factors associated with overall survival in stages 2 and 3 CRC patients: age, stage (2 vs 3), and expression of tenascin-C, OR11H1, and OR11H4 (Table 2). These factors remained significantly associated with overall survival in the multivariate analysis.

**Table 2:** Uni- and multivariate analyses of overall survival among colorectal cancer patients

	Univariate			Multivariate		
	HR	95 % CI	*p* value	HR	95 % CI	*p* value
Sex: male vs female	1.47	(0.77–2.81)	0.2397	–	–	–
Age	1.07	(1.03–1.11)	< 0.0001	1.09	(1.05–1.13)	< 0.0001
Location: right vs left	0.77	(0.39–1.54)	0.4639	–	–	–
Histologic type: MDA vs WDA	1.98	(0.77–5.05)	0.1538	–	–	–
pStage: 3 vs 2	6.03	(2.78–13.1)	< 0.0001	2.98	(1.05–8.50)	0.0410
SPP1 expression: positive vs negative	2.25	(0.95–5.36)	0.0658	–	–	–
Laminin expression: positive vs negative	1.10	(0.55–2.02)	0.7781	–	–	–
Tenascin-C expression: positive vs negative	4.75	(2.55–8.85)	<0.0001	2.16	(1.03–4.52)	0.0412
OR11H1 expression: positive vs negative	2.20	(0.86–5.61)	0.0999		–	–
OR11H4 expression: positive vs negative	5.30	(2.35–11.9)	< 0.0001	2.88	(1.10–7.56)	0.0315

Cox proportional hazards models, it is one of the statistical analysis methods we used

HR, hazard ratio; CI, confidence interval

Cox proportional hazards model

Using a similar method, we performed univariate analyses of the factors associated with disease-free survival in stages 2 and 3 CRC patients. As a result, age, stage (2 vs 3), and expression of tenascin-C, SPP1, OR11H1, and OR11H4 all were found to be associated with disease-free survival (Table 3). However, only two of these factors, age and stage (2 vs 3), remained significant in the multivariate analysis (Table 3).

**Table 3:** Uni- and multivariate analyses of disease-free survival in colorectal cancer patients

	Univariate			Multivariate		
	HR	95 % CI	*p* value	HR	95 % CI	*p* value
Sex: male vs female	1.54	(0.96–2.47)	0.0704	–	–	–
Age	1.03	(1.01–1.05)	0.0077	1.04	(1.02–1.06)	0.0004
Location: right vs left	1.01	(0.59–1.73)	0.9692	–	–	–
Histological type: MDA vs WDA	2.48	(1.19–5.15)	0.0151	–	–	–
pStage: 3 vs 2	3.51	(2.18–5.64)	< 0.0001	2.11	(1.05–4.28)	0.0367
SPP1 expression: positive vs negative	2.26	(1.22–4.17)	0.0095	1.35	(0.70–2.34)	0.3652
Laminin expression: positive vs negative	1.04	(0.63–1.72)	0.8694	–	–	–
Tenascin-C expression: positive vs negative	2.14	(1.35–3.41)	0.0012	1.12	(0.66–1.90)	0.6647
OR11H1 expression: positive vs negative	2.31	(1.22–4.38)	0.0100	1.29	(0.63–2.64)	0.4905
OR11H4 expression: positive vs negative	3.41	(2.07–5.62)	< 0.0001	1.88	(0.96–3.68)	0.0652

Cox proportional hazards models, it is one of the statistical analysis methods we used

HR, hazard ratio; CI, confidence interval

Finally, we examined the associations of clinicopathologic factors (sex, age, tumor location, histologic type, *p* stage, and overall and disease-free survival) with positive/negative expression of laminin and tenascin C. As a result, we observed no statistical differences in the clinicopathologic findings (sex, age, tumor location, histologic type, *p* stage, overall survival, or disease-free survival) in the patients with positive versus negative expression of laminin (Fig. S2). On the other hand, we observed statistical differences in the *p* stage, overall survival, and disease-free survival between those with positive and those with negative expression of tenascin-C (Fig. S2).

### Immunohistochemical Expression of Tenascin-C and OR11H1 in Primary Tumor and Metastatic Sites

We examined immunohistochemical expression of tenascin-C and OR11H1 in the primary tumor and metastatic sites (stage 3 CRC, 114 cases, Table S7). The immunohistochemical expression score of tenascin-C or OR11H1 did not differ significantly between primary and metastatic lesions (Fig. S3).

## Discussion

Metastasis of CRC, including lymph node and distant metastases, is a major issue in the clinical management of CRC.^5–7^ The presence of metastasis determines the prognosis of patients with CRC, and to improve prognosis, evaluation of the pathologic mechanism of metastasis is essential.^5–7^

This study applied an integrated bioinformatics approach to separate samples of cancer glands and the surrounding stromal tissue to determine the molecular pathways involved in CRC metastasis.^34^ Identification of target mRNAs might provide a benefit in the clinical setting for patient treatment.

Using comprehensive analyses, we found that the expression of specific mRNAs, including tenascin-C, SPP, and laminin in cancer cells, and OR11H members in CAFs is closely associated with cancer metastasis. Next, we investigated the associations between the expression of these candidate markers and the prognosis of patients with intermediate-stage (2 or 3) CRC using a large cohort. We found that upregulated expression of tenascin-C and OR11H4 was correlated with overall survival of patients with stage 2 or 3 CRC. This finding might contribute to the discovery of new diagnostic and prognostic biomarkers as well as new treatment targets for CRC. Currently, few prognostic findings are available to verify the specific mechanism associated with the novel differentially expressed genes in the CRC microenvironment.

Tenascin-C is an ECM glycoprotein that plays a crucial role in cell proliferation and tumor invasion in various cancers.^35,36^ Tenascin-C is a major protein overexpressed in both cancer cells themselves and the surrounding fibroblasts (CAFs).^36,37^Although several studies have reported that upregulated expression of tenascin-C in both cancer cells and CAFs is associated with a poor prognosis, the mechanisms whereby tenascin-C leads to shorter survival remain to be clarified in CRC.^38^

In the current study, upregulated expression of tenascin-C in cancer cells was correlated with the outcome of patients who had stage 2 or 3 CRC. This is supported by the role of tenascin-C as an important molecule promoting migration and invasion both *in vitro* and *in vivo*, and knockdown of tenascin-C in CRC cells significantly suppressed their proliferation and impaired their migration and invasion.^38^ Overall, upregulated expression of tenascin-C plays a major role in cancer progression, and metastasis and may result in a poor prognosis for CRC patients.

The metastatic potential of CRC is strongly affected by the cancer stroma.^20^ Fibroblasts, the predominant cells of the stromal cell population, are critical determinants of stromal cross-talk and cancer progression.^20–22^ A recent study showed that OR7C1, a member of the olfactory receptor family characterized by seven G protein-coupled transmembrane receptors closely associated with cancer development affects the expression of the stem cell genes SOX2, POU5F1, and LGR5 during sphere formation and tumor initiation *in vivo.*^39^ However, several olfactory receptors have been identified in tissues other than olfactory tissues, including the testis, tongue, and placenta.^39^ Hirohashi et al.^40^ showed that side population (SP) cells, which may display stem cell-like properties, derived from CRC cells have stronger tumor-initiating ability and higher expression levels of stem cell markers. In addition, SP cells are enriched among cancer-initiating cells, which develop into various differentiated progeny cells. Finally, SP cells are a reasonable source of cancer-initiating cells, which are implicated in cancer relapse and resistance to chemotherapy.^38^

In the current study, upregulated expression of OR11H4, a member of the olfactory receptor family, in stromal cells was correlated with overall survival of patients with stage 2 or 3 CRC. We suggest that OR11H4 is a novel marker predicting the outcome of patients with stage 2 or 3 CRC and may be a target of potent immunotherapies targeting cancer-initiating cells.

We were interested in comparing the tumor microenvironment between primary tumor and metastatic sites in patients who experienced development of metastatic disease.^41,42^ In the current study, the immunohistochemical expression score of tenascin-C or OR11H1 did not differ significantly between the primary tumor and metastatic sites. This result may be interesting with regard to the metastatic theory given that the expression of these proteins was retained at the metastatic site. In addition, this finding may support the hypothesis that the malignant potential of primary tumor cells is preserved at metastatic sites.

Comprehensive analysis of mRNA expression in cancer cells was performed using TCGA. Therefore, the cancer cells and surrounding stromal cells were not obtained from the same source. Although we acknowledge that more tumor samples are needed to explore the specific mRNAs expressed in cancer cells, a large public database of mRNA expression in surrounding stromal tissue is not available. In addition, study did not aim to compare gene expression between cancer and stromal cells from the same tumor. Candidate mRNAs associated with CRC metastasis that are expressed in cancer cells are considered to be independent of those expressed in stromal cells.

The cooperative role of cancer cells and the surrounding stromal cells in the tumor microenvironment was not considered in the current study. To overcome this limitation, we examined the immunohistochemical expression of the candidate proteins to identify their association with prognosis. As a result, upregulated expression of tenascin-C and OR11H4 was correlated with the prognosis of CRC patients. Immunohistochemistry also may be useful for exploring therapeutic agents for CRC given that potential mRNAs for therapeutic agents may be discovered from widely used public databases. We believe this is a reasonable approach for exploring candidate mRNAs closely associated with the prognosis of CRC patients.

The bulk sequencing data from TCGA are not ideal for comparison given that they are associated with cell populations that have abundant stromal cell contamination.^15^ Tumor content estimated pathologically may be better than that estimated molecularly.^28,29^ Indeed, contamination of stromal cells may influence target mRNA expression.^28,29^ This may be a shortcoming of TCGA, and we need to be careful when using this database. Estimation of the proportions of neoplastic and non-neoplastic cells in the cancer tissues examined may be needed to evaluate mRNA expression in cancer tissues. However, abundant sample is needed to identify candidate mRNAs associated with CRC metastasis. Given its difficulty obtaining a large tumor sample, our method of cancer gland isolation could not detect such mRNAs.^28^ For these reasons, we used TCGA to identify target mRNAs that potentially promote CRC metastasis.

This study had some limitations. First, it excluded cases of CRC with the MSI phenotype due to the different clinicopathologic and molecular features and better prognosis of MSI CRC compared with MSS CRC. Additional studies of CRC with the MSI phenotype are needed.

Second, the number of isolated stromal tissue samples was relatively small. However, it is difficult to isolate pure stromal tissue surrounding colorectal tumors. We believe the current results are novel findings useful for evaluating the molecular alterations in cancer stromal tissue.

Third, *in vivo* experimental analyses of the biologic roles of the candidate markers identified were not performed. Further examination is needed to elucidate the biologic roles of the candidate markers in metastatic CRC.

Fourth, more robust methodologies of single-cell sequencing have been used to evaluate the tumor microenvironment in a more unbiased fashion. Single-cell RNA-seq is performed to characterize transcriptomes at a cellular resolution, enabling identification of cell types and their expression profiles.^43^ This method provides the ultimate resolution and may contribute to the development of effective and personalized therapeutics for various cancers.^43^ However, single-cell sequencing is a very complex and expensive method to perform with isolated stromal cell samples. We hope to examine genome-wide expression in isolated stromal cells using this method in a future study.

Finally, we used the Clariom S array to evaluate mRNA expression, although RNA-seq often is considered a more comprehensive method for this purpose. However, RNA-seq is not always superior to microarray analysis in detecting less abundant transcripts, such as long non-coding RNAs. We believe that the Clariom S array was appropriate for examining genome-wide mRNA expression analysis in this study.

In conclusion, we explored candidate markers closely associated with CRC metastasis in both cancer tissue and the surrounding CAFs. The uni- and multivariate analyses suggested that upregulated expression of tenascin-C and OR11H4 may be useful for predicting the outcomes of patients with CRC. Further studies are needed to identify the molecular mechanisms of these gene markers in cancer progression and metastasis.

## Supplementary Information

Below is the link to the electronic supplementary material.

Supplementary file1 (DOCX 57 kb)

Supplementary file2 (DOCX 17 kb)

**Fig. S1** Kaplan–Meier analyses of overall survival based on the expression of (**a**) tenascin-C and (**b**) OR11H4. (TIF 1147 kb)

**Fig. S2 A** Sex, positivity of laminin. **B** Age distribution of laminin expression. **C** Tumor location, positivity of laminin. **D** Positivity of laminin by histologic type. **E** Positivity of laminin by stage. **F** Overall survival. positivity of laminin. **G** Disease free survival, positivity of laminin. **H** Sex, positivity of tenascin-C. **I** Age distribution of tenascin-C expression. **J** Tumor location, positivity of tenascin-C. **K** Histologic type, positivity of tenascin-C. **L** Stage, positivity of tenascin-C. **M** Overall survival, positivity of tenascin-C. **N** Disease-free survival, positivity of tenascin-C. (TIFF 289 kb)

**Fig. S3 A** Association of the immunohistochemical score of tenascin-C between the primary tumor and metastatic sites. **B** Association of the immunohistochemical score of OR11H1 between the primary tumor and metastatic sites. (TIFF 180 kb)

## Data Availability

The data supporting the findings of our study are available from the corresponding author upon reasonable request.
