# Supplementary material for: Differential Expression in the Tumor Microenvironment of mRNAs Closely Associated with Colorectal Cancer Metastasis
Source: Ann Surg Oncol. 2022 Oct 12;30(2):1255–66. doi: 10.1245/s10434-022-12574-1 (PMC9807483; doi:10.1245/s10434-022-12574-1)
Supplement: Supplementary file 1 — Supplementary file1 (DOCX 57 kb) [file 10434_2022_12574_MOESM1_ESM.docx]

Supplementary Table 1.

| Primary Antibody | Source | Clone | Dilution | Treatment |
| --- | --- | --- | --- | --- |
| SPP1 | Proteintech,  IL, USA | Polyclonal | 1:200 | None |
| Laminin | Abcam,  Cambridge, UK | Polyclonal | 1:100 | Proteinase K |
| Tenascin-C | Abcam,  Cambridge, UK | ERR4219 | 1:100 | Heat retrieval (pH9.0) |
| OR11H1 | Invitrogen,  MA, USA | Polyclonal | 1:50 | Heat retrieval (pH9.0) |
| OR11H4 | Invitrogen,  MA, USA | Polyclonal | 1:50 | Heat retrieval (pH9.0) |

Supplementary Table 2. Immunohistochemical scoring of CRC.

|  |  | Staining area (%) | | | |
| --- | --- | --- | --- | --- | --- |
|  |  | 0 (0%) | 1 (1-25%) | 2 (25-50%) | 3 (50-100%) |
| Staining intensity | 0. Negative | Score 0 | Score 0 | Score 0 | Score 0 |
|  | 1. Weak | Score 0 | Score 2 | Score 3 | Score 4 |
|  | 2. Moderate | Score 0 | Score 3 | Score 4 | Score 5 |
|  | 3. Strong | Score 0 | Score 4 | Score 5 | Score 6 |

Score 0-2: negative, Score 3-6: positive

Supplementary Table 3. Significant KEGG pathway in Gene Set Enrichment Analysis in TCGA dataset

| ID | Description | Enrichment Score | p-value |
| --- | --- | --- | --- |
| hsa04512 | ECM-receptor interaction | 0.7085 | 4.50E-08 |
| hsa04974 | Protein digestion and absorption | 0.6822 | 7.78E-07 |
| hsa04060 | Cytokine-cytokine receptor interaction | -0.4624 | 7.78E-07 |
| hsa04061 | Viral protein interaction with cytokine and cytokine receptor | -0.6354 | 7.78E-07 |
| hsa04510 | Focal adhesion | 0.5381 | 7.95E-07 |
| hsa05320 | Autoimmune thyroid disease | -0.7957 | 3.11E-06 |
| hsa05332 | Graft-versus-host disease | -0.7786 | 5.22E-06 |
| hsa05164 | Influenza A | -0.4573 | 1.60E-05 |
| hsa04612 | Antigen processing and presentation | -0.6221 | 1.60E-05 |
| hsa05330 | Allograft rejection | -0.7834 | 1.60E-05 |
| hsa01200 | Carbon metabolism | -0.5102 | 2.32E-05 |
| hsa05414 | Dilated cardiomyopathy | 0.6347 | 2.39E-05 |
| hsa05410 | Hypertrophic cardiomyopathy | 0.6281 | 3.74E-05 |
| hsa05169 | Epstein-Barr virus infection | -0.402 | 3.93E-05 |
| hsa04020 | Calcium signaling pathway | 0.5117 | 4.48E-05 |
| hsa04146 | Peroxisome | -0.5401 | 4.48E-05 |
| hsa05323 | Rheumatoid arthritis | -0.5176 | 0.0001 |
| hsa05310 | Asthma | -0.845 | 0.0001 |
| hsa01240 | Biosynthesis of cofactors | -0.4201 | 0.0002 |
| hsa04621 | NOD-like receptor signaling pathway | -0.3927 | 0.0003 |
| hsa04940 | Type I diabetes mellitus | -0.6754 | 0.0008 |
| hsa00280 | Valine, leucine and isoleucine degradation | -0.5797 | 0.0011 |
| hsa00190 | Oxidative phosphorylation | -0.4158 | 0.0012 |
| hsa05412 | Arrhythmogenic right ventricular cardiomyopathy | 0.5818 | 0.0012 |
| hsa04022 | cGMP-PKG signaling pathway | 0.4842 | 0.0019 |
| hsa04151 | PI3K-Akt signaling pathway | 0.4085 | 0.0019 |
| hsa00020 | Citrate cycle (TCA cycle) | -0.637 | 0.0031 |
| hsa05205 | Proteoglycans in cancer | 0.4392 | 0.0031 |
| hsa04062 | Chemokine signaling pathway | -0.3583 | 0.0032 |
| hsa04672 | Intestinal immune network for IgA production | -0.6352 | 0.0032 |
| hsa04360 | Axon guidance | 0.4599 | 0.0036 |
| hsa05012 | Parkinson disease | -0.3236 | 0.0054 |
| hsa04261 | Adrenergic signaling in cardiomyocytes | 0.4858 | 0.0054 |
| hsa00630 | Glyoxylate and dicarboxylate metabolism | -0.6437 | 0.0054 |
| hsa05206 | MicroRNAs in cancer | 0.4431 | 0.0062 |
| hsa04810 | Regulation of actin cytoskeleton | 0.4301 | 0.0063 |
| hsa04658 | Th1 and Th2 cell differentiation | -0.4298 | 0.0072 |
| hsa04921 | Oxytocin signaling pathway | 0.4625 | 0.0111 |
| hsa04640 | Hematopoietic cell lineage | -0.4388 | 0.0138 |
| hsa04659 | Th17 cell differentiation | -0.3878 | 0.0168 |
| hsa00270 | Cysteine and methionine metabolism | -0.519 | 0.0172 |
| hsa01230 | Biosynthesis of amino acids | -0.4448 | 0.0172 |
| hsa00240 | Pyrimidine metabolism | -0.4753 | 0.0172 |
| hsa04270 | Vascular smooth muscle contraction | 0.4734 | 0.0174 |
| hsa04926 | Relaxin signaling pathway | 0.4548 | 0.0181 |
| hsa04014 | Ras signaling pathway | 0.4083 | 0.0184 |
| hsa00071 | Fatty acid degradation | -0.5129 | 0.0184 |
| hsa00970 | Aminoacyl-tRNA biosynthesis | -0.4897 | 0.019 |
| hsa05150 | Staphylococcus aureus infection | -0.4693 | 0.02 |
| hsa05014 | Amyotrophic lateral sclerosis | -0.2713 | 0.0208 |
| hsa00620 | Pyruvate metabolism | -0.5055 | 0.0232 |
| hsa05032 | Morphine addiction | 0.5111 | 0.0239 |
| hsa00910 | Nitrogen metabolism | -0.7769 | 0.0247 |
| hsa04623 | Cytosolic DNA-sensing pathway | -0.4795 | 0.0248 |
| hsa04650 | Natural killer cell mediated cytotoxicity | -0.387 | 0.0291 |
| hsa00860 | Porphyrin metabolism | -0.5322 | 0.0293 |
| hsa04010 | MAPK signaling pathway | 0.3717 | 0.0318 |
| hsa04713 | Circadian entrainment | 0.5017 | 0.0318 |
| hsa00232 | Caffeine metabolism | -0.9485 | 0.0318 |
| hsa00983 | Drug metabolism - other enzymes | -0.468 | 0.0318 |
| hsa04371 | Apelin signaling pathway | 0.4393 | 0.0318 |
| hsa05321 | Inflammatory bowel disease | -0.4633 | 0.0385 |
| hsa03010 | Ribosome | -0.3275 | 0.0396 |
| hsa01212 | Fatty acid metabolism | -0.4411 | 0.0457 |
| hsa05165 | Human papillomavirus infection | 0.3582 | 0.0475 |

Supplementary Table 4. Expression of genes contained in ECM-receptor interaction pathway (KEGG entry hsa04512) in the TCGA dataset.

| Gene symbol | Fold Change | p-value |
| --- | --- | --- |
| SPP1 | 1.767 | 0.0074 |
| THBS4 | 1.666 | 0.0362 |
| COL9A3 | 1.526 | 0.0253 |
| FREM1 | 1.48 | 0.0442 |
| TNXB | 1.364 | 0.0655 |
| COMP | 1.311 | 0.2899 |
| ITGA7 | 1.276 | 0.0193 |
| VWF | 1.259 | 0.0422 |
| ITGA5 | 1.252 | 0.0432 |
| SV2A | 1.251 | 0.0532 |
| CD36 | 1.248 | 0.1851 |
| THBS2 | 1.239 | 0.2667 |
| LAMA5 | 1.233 | 0.0885 |
| COL6A3 | 1.222 | 0.1563 |
| COL4A2 | 1.22 | 0.0282 |
| COL4A4 | 1.212 | 0.3002 |
| ITGA3 | 1.201 | 0.0256 |
| TNC | 1.198 | 0.2886 |
| COL1A2 | 1.196 | 0.3472 |
| COL4A1 | 1.188 | 0.0669 |
| FREM2 | 1.182 | 0.4945 |
| ITGB3 | 1.182 | 0.1526 |
| THBS3 | 1.178 | 0.0416 |
| LAMA2 | 1.173 | 0.3334 |
| LAMB3 | 1.163 | 0.0556 |
| LAMC2 | 1.158 | 0.1018 |
| LAMA4 | 1.144 | 0.1496 |
| THBS1 | 1.144 | 0.277 |
| COL9A1 | 1.142 | 0.5216 |
| AGRN | 1.141 | 0.1406 |
| COL1A1 | 1.14 | 0.569 |
| LAMC1 | 1.14 | 0.0438 |
| COL6A2 | 1.136 | 0.379 |
| LAMB2 | 1.136 | 0.1554 |
| FRAS1 | 1.133 | 0.5263 |
| FN1 | 1.132 | 0.5571 |
| HSPG2 | 1.126 | 0.3215 |
| ITGA11 | 1.109 | 0.5599 |
| COL6A1 | 1.104 | 0.4756 |
| ITGA1 | 1.098 | 0.2637 |
| ITGB5 | 1.094 | 0.1496 |
| SDC4 | 1.088 | 0.2827 |
| ITGB1 | 1.083 | 0.2317 |
| LAMC3 | 1.071 | 0.6982 |
| ITGAV | 1.068 | 0.4984 |
| ITGA9 | 1.055 | 0.7209 |
| SDC1 | 1.048 | 0.5085 |
| ITGB6 | 1.042 | 0.693 |
| ITGB4 | 1.029 | 0.7977 |
| ITGA4 | 1.024 | 0.8881 |
| ITGA8 | 1.014 | 0.9364 |
| LAMB1 | 1.006 | 0.9643 |
| ITGB8 | -1 | 0.9995 |
| COL9A2 | -1.014 | 0.9582 |
| CD47 | -1.022 | 0.7848 |
| ITGA2 | -1.028 | 0.7869 |
| NPNT | -1.032 | 0.8588 |
| LAMA3 | -1.041 | 0.7923 |
| DAG1 | -1.053 | 0.3496 |
| HMMR | -1.053 | 0.6568 |
| COL4A5 | -1.064 | 0.7754 |
| ITGA6 | -1.082 | 0.2945 |
| CD44 | -1.099 | 0.1235 |
| ITGB7 | -1.11 | 0.5306 |
| LAMA1 | -1.184 | 0.2738 |
| COL2A1 | #N/A | #N/A |
| COL4A6 | #N/A | #N/A |
| COL4A3 | #N/A | #N/A |
| COL6A6 | #N/A | #N/A |
| COL6A5 | #N/A | #N/A |
| LAMB4 | #N/A | #N/A |
| CHAD | #N/A | #N/A |
| RELN | #N/A | #N/A |
| VTN | #N/A | #N/A |
| TNN | #N/A | #N/A |
| TNR | #N/A | #N/A |
| DSPP | #N/A | #N/A |
| IBSP | #N/A | #N/A |
| DMP1 | #N/A | #N/A |
| ITGA2B | #N/A | #N/A |
| ITGA10 | #N/A | #N/A |
| SV2C | #N/A | #N/A |
| SV2B | #N/A | #N/A |
| GP5 | #N/A | #N/A |
| GP1BA | #N/A | #N/A |
| GP1BB | #N/A | #N/A |
| GP9 | #N/A | #N/A |
| GP6 | #N/A | #N/A |

Supplementary Table 5. Significant KEGG pathway in Gene Set Enrichment Analysis in isolated cancer stroma

| ID | Description | Enrichment Score | p-value |
| --- | --- | --- | --- |
| hsa04740 | Olfactory transduction | 0.1224 | 0.0104 |
| hsa05161 | Hepatitis B | -0.1809 | 0.0134 |
| hsa05330 | Allograft rejection | -0.3614 | 0.0144 |
| hsa04613 | Neutrophil extracellular trap formation | -0.1693 | 0.0144 |
| hsa04141 | Protein processing in endoplasmic reticulum | -0.1727 | 0.0144 |
| hsa05203 | Viral carcinogenesis | -0.1575 | 0.0144 |
| hsa04940 | Type I diabetes mellitus | -0.3257 | 0.0144 |
| hsa00130 | Ubiquinone and other terpenoid-quinone biosynthesis | -0.6032 | 0.0144 |
| hsa04218 | Cellular senescence | -0.1728 | 0.0256 |
| hsa05321 | Inflammatory bowel disease | -0.2507 | 0.0308 |
| hsa05415 | Diabetic cardiomyopathy | -0.1488 | 0.0308 |
| hsa05322 | Systemic lupus erythematosus | -0.1774 | 0.0323 |

Supplementary Table 6. Expression of genes contained in the olfactory receptor (KEGG entry K04257) in the isolated cancer stroma.

| Gene symbol | Fold change | p-value |
| --- | --- | --- |
| OR52A5 | 2.033 | 0.0072 |
| OR13C4 | 1.894 | 0.1525 |
| OR11H4 | 1.826 | 0.0005 |
| OR11H1 | 1.692 | 0.0056 |
| OR4K1 | 1.615 | 0.0336 |
| OR8K3 | 1.613 | 0.0039 |
| OR9Q2 | 1.6 | 0.0436 |
| OR8B3 | 1.562 | 0.0557 |
| OR1L3 | 1.55 | 0.0009 |
| OR10A6 | 1.541 | 0.0141 |
| OR2F2 | 1.511 | 0.074 |
| OR2B3 | 1.51 | 0.017 |
| OR5AS1 | 1.507 | 0.0216 |
| OR4L1 | 1.498 | 0.0235 |
| OR4Q3 | 1.473 | 0.2194 |
| OR8D2 | 1.47 | 0.0578 |
| OR9I1 | 1.459 | 0.0514 |
| OR4P4 | 1.442 | 0.5426 |
| OR52N1 | 1.42 | 0.0766 |
| OR8B2 | 1.407 | 0.4284 |
| OR5D13 | 1.4 | 0.0262 |
| OR11H2 | 1.396 | 0.0729 |
| OR5AL1 | 1.385 | 0.1757 |
| OR11H7 | 1.38 | 0.1095 |
| OR6C75 | 1.379 | 0.3142 |
| OR6C1 | 1.379 | 0.0478 |
| OR8H3 | 1.369 | 0.0258 |
| OR51A7 | 1.352 | 0.1524 |
| OR4K17 | 1.343 | 0.0241 |
| OR9A2 | 1.338 | 0.0188 |
| OR6C74 | 1.31 | 0.0247 |
| OR4Q2 | 1.299 | 0.1199 |
| OR1L8 | 1.295 | 0.0078 |
| OR4S2 | 1.29 | 0.1933 |
| OR10A3 | 1.289 | 0.035 |
| OR5B17 | 1.279 | 0.082 |
| OR5F1 | 1.278 | 0.1459 |
| OR4C11 | 1.27 | 0.316 |
| OR13C3 | 1.266 | 0.1579 |
| OR1C1 | 1.264 | 0.1807 |
| OR8G5 | 1.263 | 0.0452 |
| OR10A7 | 1.261 | 0.0501 |
| OR8D1 | 1.26 | 0.0414 |
| OR10J1 | 1.258 | 0.1175 |
| OR4A16 | 1.255 | 0.1287 |
| OR13G1 | 1.251 | 0.2508 |
| OR5I1 | 1.248 | 0.1374 |
| OR13H1 | 1.239 | 0.1609 |
| OR6C68 | 1.232 | 0.1149 |
| OR6M1 | 1.231 | 0.0323 |
| OR4K5 | 1.223 | 0.2452 |
| OR9K2 | 1.221 | 0.4248 |
| OR10S1 | 1.217 | 0.1623 |
| OR5K4 | 1.213 | 0.218 |
| OR5K3 | 1.21 | 0.0369 |
| OR4N2 | 1.202 | 0.0436 |
| OR12D3 | 1.199 | 0.2079 |
| OR10C1 | 1.198 | 0.0988 |
| OR8J3 | 1.198 | 0.4691 |
| OR8I2 | 1.198 | 0.5033 |
| OR5T2 | 1.196 | 0.0082 |
| OR11H6 | 1.196 | 0.2002 |
| OR51B6 | 1.193 | 0.3784 |
| OR51G1 | 1.192 | 0.089 |
| OR10AG1 | 1.19 | 0.0572 |
| OR2AG1 | 1.187 | 0.2756 |
| OR52D1 | 1.184 | 0.8821 |
| OR5AN1 | 1.183 | 0.3304 |
| OR12D2 | 1.183 | 0.2322 |
| OR10A4 | 1.18 | 0.9877 |
| OR4C45 | 1.179 | 0.1363 |
| OR52N4 | 1.177 | 0.0399 |
| OR4C12 | 1.173 | 0.1839 |
| OR1Q1 | 1.169 | 0.3583 |
| OR9A4 | 1.164 | 0.317 |
| OR52K2 | 1.164 | 0.2476 |
| OR8H1 | 1.163 | 0.0626 |
| OR1B1 | 1.163 | 0.3339 |
| OR13F1 | 1.163 | 0.3512 |
| OR4K14 | 1.163 | 0.5525 |
| OR10A5 | 1.161 | 0.3232 |
| OR1E1 | 1.159 | 0.2955 |
| OR4D10 | 1.158 | 0.3637 |
| OR5AK2 | 1.157 | 0.6098 |
| OR1L4 | 1.153 | 0.2794 |
| OR6B1 | 1.152 | 0.0854 |
| OR5H1 | 1.152 | 0.1564 |
| OR10G2 | 1.145 | 0.2008 |
| OR51I2 | 1.144 | 0.821 |
| OR5M3 | 1.144 | 0.3011 |
| OR4K15 | 1.142 | 0.3005 |
| OR5J2 | 1.14 | 0.2867 |
| OR5K2 | 1.139 | 0.1866 |
| OR5T1 | 1.139 | 0.4805 |
| OR6C3 | 1.137 | 0.1424 |
| OR52B2 | 1.133 | 0.3067 |
| OR51M1 | 1.129 | 0.3087 |
| OR10G8 | 1.129 | 0.2018 |
| OR14J1 | 1.125 | 0.1706 |
| OR10G7 | 1.123 | 0.0989 |
| OR1J2 | 1.121 | 0.393 |
| OR5P2 | 1.121 | 0.2427 |
| OR2Y1 | 1.12 | 0.4191 |
| OR51A4 | 1.119 | 0.9519 |
| OR52E6 | 1.118 | 0.2331 |
| OR4F6 | 1.114 | 0.6607 |
| OR13D1 | 1.113 | 0.9381 |
| OR7D4 | 1.111 | 0.3527 |
| OR52W1 | 1.108 | 0.1333 |
| OR2C1 | 1.108 | 0.4623 |
| OR4C13 | 1.108 | 0.5181 |
| OR2G2 | 1.106 | 0.5287 |
| OR10K2 | 1.106 | 0.3168 |
| OR56A1 | 1.104 | 0.8655 |
| OR11H12 | 1.103 | 0.3119 |
| OR52J3 | 1.101 | 0.4097 |
| OR51T1 | 1.097 | 0.8866 |
| OR5L2 | 1.095 | 0.3535 |
| OR4M1 | 1.095 | 0.7144 |
| OR6C2 | 1.095 | 0.4369 |
| OR52M1 | 1.093 | 0.607 |
| OR4D9 | 1.092 | 0.3904 |
| OR1A1 | 1.091 | 0.4928 |
| OR1S2 | 1.091 | 0.1473 |
| OR9G4 | 1.091 | 0.152 |
| OR2V2 | 1.09 | 0.989 |
| OR2M3 | 1.088 | 0.6044 |
| OR2AT4 | 1.088 | 0.1678 |
| OR4D5 | 1.086 | 0.3653 |
| OR5V1 | 1.086 | 0.2722 |
| OR14C36 | 1.084 | 0.6223 |
| OR2AP1 | 1.081 | 0.4977 |
| OR9Q1 | 1.079 | 0.0739 |
| OR2G3 | 1.079 | 0.9307 |
| OR5M11 | 1.076 | 0.7018 |
| OR51J1 | 1.076 | 0.39 |
| OR52N2 | 1.076 | 0.5152 |
| OR8G1 | 1.076 | 0.5005 |
| OR4S1 | 1.074 | 0.5703 |
| OR2H1 | 1.073 | 0.4402 |
| OR56A4 | 1.073 | 0.353 |
| OR2A12 | 1.073 | 0.5536 |
| OR56B4 | 1.072 | 0.3855 |
| OR6N1 | 1.07 | 0.5928 |
| OR8B12 | 1.069 | 0.3449 |
| OR4C15 | 1.069 | 0.4875 |
| OR10AD1 | 1.068 | 0.333 |
| OR1S1 | 1.067 | 0.0742 |
| OR51G2 | 1.067 | 0.9275 |
| OR2W3 | 1.065 | 0.3821 |
| OR8K5 | 1.065 | 0.4729 |
| OR5T3 | 1.063 | 0.9813 |
| OR4D11 | 1.062 | 0.7337 |
| OR10J5 | 1.06 | 0.4041 |
| OR8J1 | 1.055 | 0.7023 |
| OR51V1 | 1.054 | 0.1236 |
| OR2AG2 | 1.054 | 0.782 |
| OR2A25 | 1.053 | 0.6261 |
| OR3A3 | 1.052 | 0.4897 |
| OR2T33 | 1.049 | 0.9192 |
| OR10A2 | 1.048 | 0.4672 |
| OR2L8 | 1.048 | 0.4762 |
| OR2J3 | 1.047 | 0.8761 |
| OR5K1 | 1.046 | 0.7206 |
| OR1D5 | 1.045 | 0.8007 |
| OR11A1 | 1.043 | 0.2941 |
| OR5A2 | 1.041 | 0.6734 |
| OR2T3 | 1.041 | 0.6138 |
| OR2L13 | 1.041 | 0.4617 |
| OR8H2 | 1.04 | 0.4452 |
| OR6S1 | 1.039 | 0.4966 |
| OR4C16 | 1.037 | 0.8535 |
| OR4C6 | 1.036 | 0.2767 |
| OR4D2 | 1.035 | 0.3915 |
| OR2T35 | 1.034 | 0.9869 |
| OR4C3 | 1.033 | 0.7569 |
| OR6X1 | 1.031 | 0.237 |
| OR6A2 | 1.031 | 0.3636 |
| OR51F1 | 1.031 | 0.7825 |
| OR52E1 | 1.027 | 0.898 |
| OR4F5 | 1.026 | 0.5603 |
| OR2D3 | 1.025 | 0.4709 |
| OR3A2 | 1.025 | 0.8667 |
| OR6C6 | 1.023 | 0.6043 |
| OR7C1 | 1.023 | 0.8116 |
| OR52B4 | 1.02 | 0.8041 |
| OR6C76 | 1.02 | 0.8793 |
| OR4N4 | 1.019 | 0.6022 |
| OR2G6 | 1.019 | 0.6238 |
| OR4X2 | 1.018 | 0.5858 |
| OR2M7 | 1.017 | 0.8358 |
| OR5M10 | 1.016 | 0.2475 |
| OR2B6 | 1.016 | 0.4403 |
| OR4A47 | 1.016 | 0.8505 |
| OR6C70 | 1.015 | 0.503 |
| OR1L1 | 1.015 | 0.7631 |
| OR2T12 | 1.014 | 0.3308 |
| OR5AR1 | 1.014 | 0.6488 |
| OR56A3 | 1.013 | 0.8185 |
| OR2T34 | 1.011 | 0.7198 |
| OR10K1 | 1.006 | 0.9114 |
| OR13C5 | 1.005 | 0.8713 |
| OR51B5 | 1.005 | 0.99 |
| OR1E2 | 1.005 | 0.6971 |
| OR10Z1 | 1.004 | 0.3792 |
| OR6K3 | 1.004 | 0.94 |
| OR10G9 | 1.003 | 0.5273 |
| OR8D4 | 1.001 | 0.7215 |
| OR13A1 | 1.001 | 0.9038 |
| OR4A5 | -1.001 | 0.4075 |
| OR51B2 | -1.002 | 0.7754 |
| OR2B2 | -1.002 | 0.2589 |
| OR56B1 | -1.007 | 0.3327 |
| OR5W2 | -1.008 | 0.9624 |
| OR10G4 | -1.008 | 0.8038 |
| OR51E2 | -1.008 | 0.9602 |
| OR51D1 | -1.009 | 0.6075 |
| OR1L6 | -1.014 | 0.6741 |
| OR51Q1 | -1.015 | 0.9723 |
| OR10T2 | -1.016 | 0.7135 |
| OR5M1 | -1.018 | 0.9017 |
| OR11G2 | -1.018 | 0.7368 |
| OR2T8 | -1.02 | 0.9892 |
| OR6Q1 | -1.02 | 0.4465 |
| OR52B6 | -1.02 | 0.8091 |
| OR4A15 | -1.026 | 0.7285 |
| OR2B11 | -1.028 | 0.5346 |
| OR52N5 | -1.029 | 0.822 |
| OR13J1 | -1.029 | 0.6153 |
| OR52A1 | -1.031 | 0.9432 |
| OR1D2 | -1.031 | 0.8577 |
| OR1D4 | -1.031 | 0.8693 |
| OR10W1 | -1.032 | 0.7555 |
| OR5D14 | -1.033 | 0.9964 |
| OR52E8 | -1.034 | 0.7919 |
| OR1A2 | -1.034 | 0.5673 |
| OR2J1 | -1.036 | 0.6451 |
| OR52E2 | -1.037 | 0.624 |
| OR4D1 | -1.042 | 0.3918 |
| OR5B21 | -1.043 | 0.5729 |
| OR6Y1 | -1.047 | 0.8765 |
| OR1M1 | -1.05 | 0.5865 |
| OR51E1 | -1.053 | 0.8889 |
| OR5AU1 | -1.053 | 0.2703 |
| OR2M2 | -1.053 | 0.5421 |
| OR52I2 | -1.059 | 0.9575 |
| OR5A1 | -1.06 | 0.7492 |
| OR4F17 | -1.061 | 0.8234 |
| OR6P1 | -1.061 | 0.803 |
| OR52I1 | -1.064 | 0.8444 |
| OR6N2 | -1.065 | 0.8183 |
| OR2V1 | -1.066 | 0.4484 |
| OR51B4 | -1.066 | 0.4515 |
| OR2T5 | -1.068 | 0.4679 |
| OR52H1 | -1.068 | 0.2738 |
| OR7A5 | -1.068 | 0.768 |
| OR5B12 | -1.069 | 0.8936 |
| OR5H14 | -1.069 | 0.7186 |
| OR51S1 | -1.07 | 0.6282 |
| OR5B3 | -1.07 | 0.7147 |
| OR4K2 | -1.071 | 0.6599 |
| OR2M4 | -1.073 | 0.8845 |
| OR4B1 | -1.075 | 0.7758 |
| OR4F15 | -1.076 | 0.8493 |
| OR6C65 | -1.079 | 0.5398 |
| OR10H2 | -1.079 | 0.3336 |
| OR1K1 | -1.081 | 0.6697 |
| OR6T1 | -1.086 | 0.8921 |
| OR2H2 | -1.088 | 0.4303 |
| OR52E4 | -1.092 | 0.7006 |
| OR6J1 | -1.095 | 0.7235 |
| OR6B3 | -1.098 | 0.5522 |
| OR2T4 | -1.101 | 0.6549 |
| OR8K1 | -1.101 | 0.5229 |
| OR4N5 | -1.103 | 0.6126 |
| OR52K1 | -1.104 | 0.3329 |
| OR5AP2 | -1.105 | 0.5947 |
| OR4K13 | -1.108 | 0.6023 |
| OR4C46 | -1.108 | 0.6821 |
| OR2S2 | -1.109 | 0.1676 |
| OR11L1 | -1.115 | 0.411 |
| OR1N1 | -1.116 | 0.4832 |
| OR2D2 | -1.118 | 0.8342 |
| OR2AK2 | -1.121 | 0.9922 |
| OR6K6 | -1.122 | 0.677 |
| OR2T6 | -1.122 | 0.3153 |
| OR2K2 | -1.124 | 0.6484 |
| OR7E24 | -1.126 | 0.6144 |
| OR7D2 | -1.126 | 0.5322 |
| OR1F1 | -1.132 | 0.1547 |
| OR4M2 | -1.135 | 0.6383 |
| OR10R2 | -1.135 | 0.1539 |
| OR10H5 | -1.138 | 0.2052 |
| OR8S1 | -1.138 | 0.2783 |
| OR4E2 | -1.138 | 0.7674 |
| OR10Q1 | -1.138 | 0.0895 |
| OR13C8 | -1.141 | 0.9103 |
| OR2T11 | -1.147 | 0.3317 |
| OR8A1 | -1.147 | 0.7513 |
| OR52L1 | -1.149 | 0.4458 |
| OR2T27 | -1.15 | 0.2668 |
| OR2T10 | -1.15 | 0.3122 |
| OR5M8 | -1.151 | 0.451 |
| OR2A4 | -1.152 | 0.4472 |
| OR10P1 | -1.157 | 0.0977 |
| OR5L1 | -1.157 | 0.1478 |
| OR5M9 | -1.16 | 0.1212 |
| OR2A5 | -1.164 | 0.1727 |
| OR56A5 | -1.166 | 0.2835 |
| OR6K2 | -1.17 | 0.1894 |
| OR4C5 | -1.175 | 0.2282 |
| OR2L2 | -1.176 | 0.9895 |
| OR14I1 | -1.182 | 0.4339 |
| OR14A16 | -1.187 | 0.4761 |
| OR1I1 | -1.188 | 0.0713 |
| OR2T29 | -1.188 | 0.2544 |
| OR4F21 | -1.195 | 0.6225 |
| OR5H6 | -1.198 | 0.9003 |
| OR5C1 | -1.201 | 0.0452 |
| OR2T2 | -1.206 | 0.1918 |
| OR6B2 | -1.206 | 0.2861 |
| OR52R1 | -1.207 | 0.3344 |
| OR2AE1 | -1.211 | 0.1411 |
| OR2L5 | -1.214 | 0.7073 |
| OR10X1 | -1.218 | 0.0877 |
| OR2W1 | -1.218 | 0.2701 |
| OR2L3 | -1.222 | 0.2482 |
| OR10H4 | -1.223 | 0.0731 |
| OR2T1 | -1.228 | 0.0349 |
| OR4D6 | -1.228 | 0.6571 |
| OR13C2 | -1.232 | 0.1737 |
| OR4X1 | -1.237 | 0.1076 |
| OR5AC2 | -1.238 | 0.0445 |
| OR8B4 | -1.242 | 0.1295 |
| OR5D18 | -1.246 | 0.5314 |
| OR51L1 | -1.248 | 0.6098 |
| OR4F3 | -1.251 | 0.1487 |
| OR7A17 | -1.255 | 0.0949 |
| OR1J1 | -1.257 | 0.0779 |
| OR7C2 | -1.259 | 0.0594 |
| OR2F1 | -1.262 | 0.0623 |
| OR2J2 | -1.264 | 0.5425 |
| OR10H3 | -1.273 | 0.1154 |
| OR10V1 | -1.3 | 0.1102 |
| OR8B8 | -1.309 | 0.082 |
| OR5D16 | -1.335 | 0.2969 |
| OR10H1 | -1.342 | 0.1366 |
| OR3A1 | -1.345 | 0.3401 |
| OR5P3 | -1.349 | 0.034 |
| OR6V1 | -1.355 | 0.002 |
| OR6C4 | -1.356 | 0.1952 |
| OR13C9 | -1.369 | 0.0321 |
| OR5B2 | -1.381 | 0.0344 |
| OR1G1 | -1.408 | 0.1815 |
| OR7A10 | -1.412 | 0.0101 |
| OR7G3 | -1.447 | 0.0037 |
| OR5H2 | -1.458 | 0.1003 |
| OR2M5 | -1.459 | 0.014 |
| OR7G2 | -1.464 | 0.0988 |
| OR51I1 | -1.532 | 0.0088 |
| OR2Z1 | -1.577 | 0.0052 |
| OR10G3 | -1.595 | 0.0232 |
| OR2A1 | -1.598 | 0.0376 |
| OR51F2 | -1.647 | 0.0077 |
| OR1N2 | -1.677 | 0.0008 |
| OR51A2 | -1.723 | 0.3158 |
| OR7G1 | -1.734 | 0.0001 |
| OR1J4 | -1.771 | 0.0004 |
| OR4N4C | #N/A | #N/A |
| OR2AJ1 | #N/A | #N/A |
| OR2A14 | #N/A | #N/A |
| OR8U1 | #N/A | #N/A |
| OR8U3 | #N/A | #N/A |
| OR10D3 | #N/A | #N/A |
| OR12D1 | #N/A | #N/A |
| OR4E1 | #N/A | #N/A |
| OR4K3 | #N/A | #N/A |
| OR6F1 | #N/A | #N/A |
| OR14K1 | #N/A | #N/A |
| OR14A2 | #N/A | #N/A |
| OR52E5 | #N/A | #N/A |
| OR9G1 | #N/A | #N/A |
| OR10J4 | #N/A | #N/A |
| OR10AC1 | #N/A | #N/A |
| OR2A7 | #N/A | #N/A |
| OR2A42 | #N/A | #N/A |
| OR5H15 | #N/A | #N/A |
| OR2A2 | #N/A | #N/A |
| OR8U8 | #N/A | #N/A |
| OR8U9 | #N/A | #N/A |
| OR9G9 | #N/A | #N/A |
| OR4F29 | #N/A | #N/A |
| OR10G6 | #N/A | #N/A |
| OR4F16 | #N/A | #N/A |
| OR2T7 | #N/A | #N/A |
| OR2C3 | #N/A | #N/A |

Supplementary Table 7. Clinicopathological findings in validation cohort

|  | Cases (%) | No metastasis (%) | Metastasis (%) | p-value |
| --- | --- | --- | --- | --- |
| **Total** | 259 | 145 | 114 |  |
| **Sex** |  |  |  |  |
| Male | 153 (56.0) | 79 (54.5) | 74 (64.9) |  |
| Female | 106 (44.0) | 66 (45.5) | 40 (35.1) | N.S. |
| Age, years,  median [range] | 69 [34–92] | 70 [34–88] | 68 [41–92] | N.S. |
| **Location** |  |  |  |  |
| Right side | 56 (21.6) | 32 (22.1) | 24 (21.1) |  |
| Left side | 203 (78.4) | 113 (77.9) | 90 (78.9) | N.S. |
| **Histological type** |  |  |  |  |
| WDA | 50 (19.3) | 36 (24.8) | 14 (12.3) |  |
| MDA | 209 (80.7) | 109 (75.2) | 100 (87.7) | <0.05 |
| **Stage** |  |  |  | <0.0001 |
| I | 0 (0) | 0 (0) | 0 |  |
| II | 145 (56.0) | 145 (100) | 0 |  |
| III | 114 (44.0) | 0 (0) | 114 (100) |  |
| IV | 0 (0) | 0 (0) | 0 |  |
| OS, days, median [range] | 2093  [52–3860] | 2112  [93–3734] | 1917.5  [52–3860] | <0.05 |
| DFS, days,  median [range] | 1825  [33–3734] | 1862  [84–3734] | 1191  [33–3688] | <0.0001 |

WDA: well-differentiated adenocarcinoma; MDA: moderately differentiated adenocarcinoma; OS: overall survival; DFS: disease-free survival; N.S.: not significant.
