# Supplementary material for: Differential Expression in the Tumor Microenvironment of mRNAs Closely Associated with Colorectal Cancer Metastasis
Source: Ann Surg Oncol. 2022 Oct 12;30(2):1255–66. doi: 10.1245/s10434-022-12574-1 (PMC9807483; doi:10.1245/s10434-022-12574-1)
Supplement: Supplementary file 2 — Supplementary file2 (DOCX 17 kb) [file 10434_2022_12574_MOESM2_ESM.docx]

**Supplementary methods**

**RNA and DNA extraction**

mRNA was extracted from the surrounding stromal tissue using the mirVana™ miRNA Isolation kit (Thermo Fisher Scientific, Inc.) according to the manufacturer's instructions. RNA quantity and quality were evaluated using the DU730 spectrophotometer (Beckman Coulter, Brea, CA, USA), and RNA integrity was determined by gel electrophoresis.

For determination of the microsatellite status of cancer glands, DNA from normal and tumor glands was extracted by standard SDS proteinase K treatment. DNA extracted from the samples was resuspended in TE buffer (10 mM Tris-HCl, 1 mM EDTA [pH 8.0]).

**Analysis of microsatellite instability (MSI)**

See the Supplementary Methods for details regarding the DNA extraction method. The MSI status was determined using a consensus panel including five reference microsatellite markers (BAT25, BAT26, D2S123, D3S546, and D17S250), as described previously.30 The criteria used to define MSI were described previously. In brief, when no marker was altered, the tumors were defined as MSS. When only one marker was altered, the tumors were defined as low MSI. When two or more markers were altered, the tumors were defined as high MSI.

**Immunohistochemistry**

We examined candidate markers potentially associated with CRC metastasis using immunohistochemistry (the candidate markers were obtained based on data from the first and second cohorts). Tumors were fixed in 20% neutral-buffered formalin and embedded in paraffin wax. Paraffin sections (3 µm thick) were cut, dewaxed, and rehydrated. Microarray slides were incubated in 3% hydrogen peroxide to block endogenous peroxidase. Antigen retrieval was performed using an autoclave-based method, followed by incubation with the primary antibody overnight at 4°C in a high-humidity cabinet. Slides were processed using the Dako Autostainer Universal Staining System (Dako, Glostrup, Denmark). The specimens were treated with citrate buffer (pH 6.0) using a microwave (three times for 5 min, 750 W; cat. no. H2500; Microwave Processor [Bio-Rad Laboratories, CA, USA]) and then reacted with the antibodies, as described previously. The antibodies used in this study targeted markers of either cancer cells (tenascin-C, laminin, and secreted phosphoprotein 1 [SPP1]) or CAFs (OR11H1 and OR11H4). Detailed information about the antibodies is summarized in **Supplementary Table 1**.
