# Supplementary figures and images for: Differential Expression in the Tumor Microenvironment of mRNAs Closely Associated with Colorectal Cancer Metastasis
Source: Ann Surg Oncol. 2022 Oct 12;30(2):1255–66. doi: 10.1245/s10434-022-12574-1 (PMC9807483; doi:10.1245/s10434-022-12574-1)

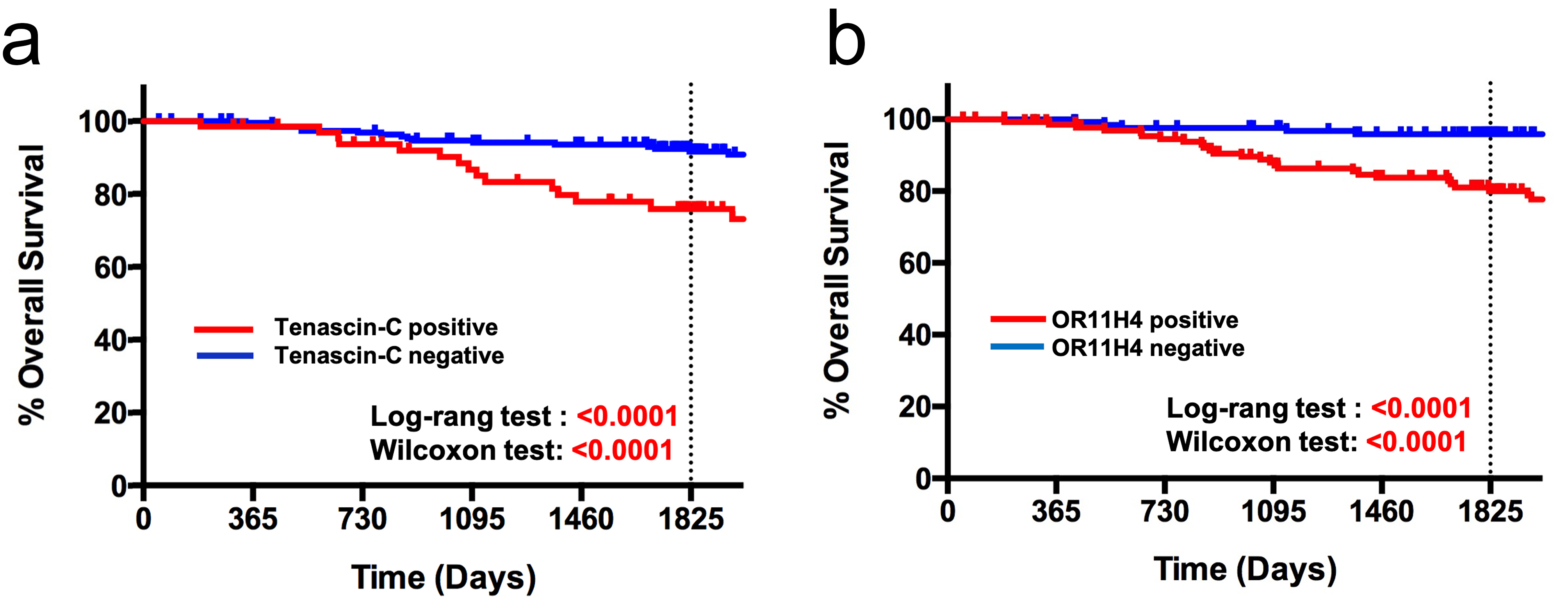

Supplement: Supplementary file 3 — Fig. S1 Kaplan–Meier analyses of overall survival based on the expression of (a) tenascin-C and (b) OR11H4. (TIF 1147 kb) [file 10434_2022_12574_MOESM3_ESM.tif]

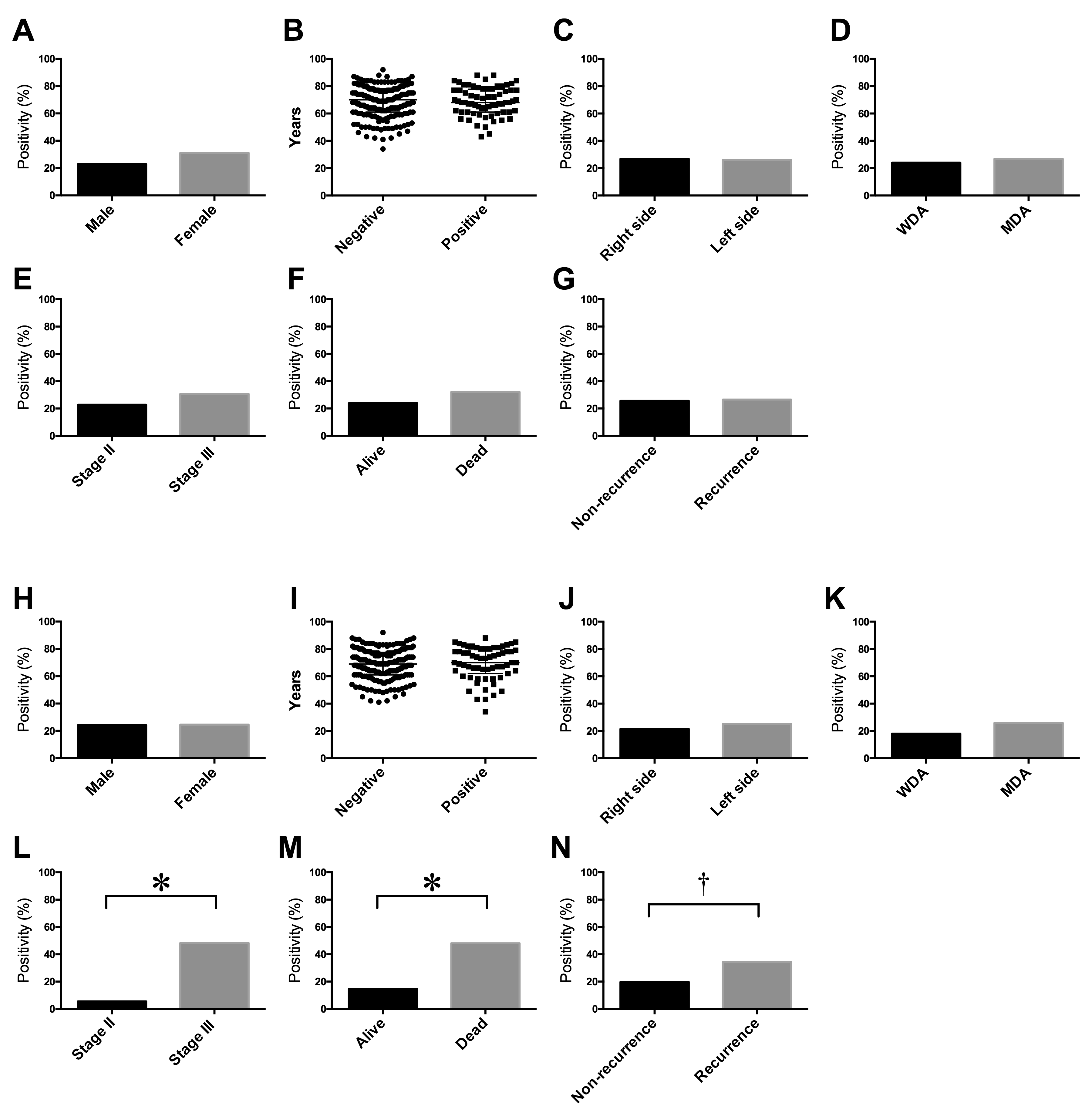

Supplement: Supplementary file 4 — Fig. S2 A Sex, positivity of laminin. B Age distribution of laminin expression. C Tumor location, positivity of laminin. D Positivity of laminin by histologic type. E Positivity of laminin by stage. F Overall survival. positivity of laminin. G Disease free survival, positivity of laminin. H Sex, positivity of tenascin-C. I Age distribution of tenascin-C expression. J Tumor location, positivity of tenascin-C. K Histologic type, positivity of tenascin-C. L Stage, positivity of tenascin-C. M Overall survival, positivity of tenascin-C. N Disease-free survival, positivity of tenascin-C. (TIFF 289 kb) [file 10434_2022_12574_MOESM4_ESM.tiff]

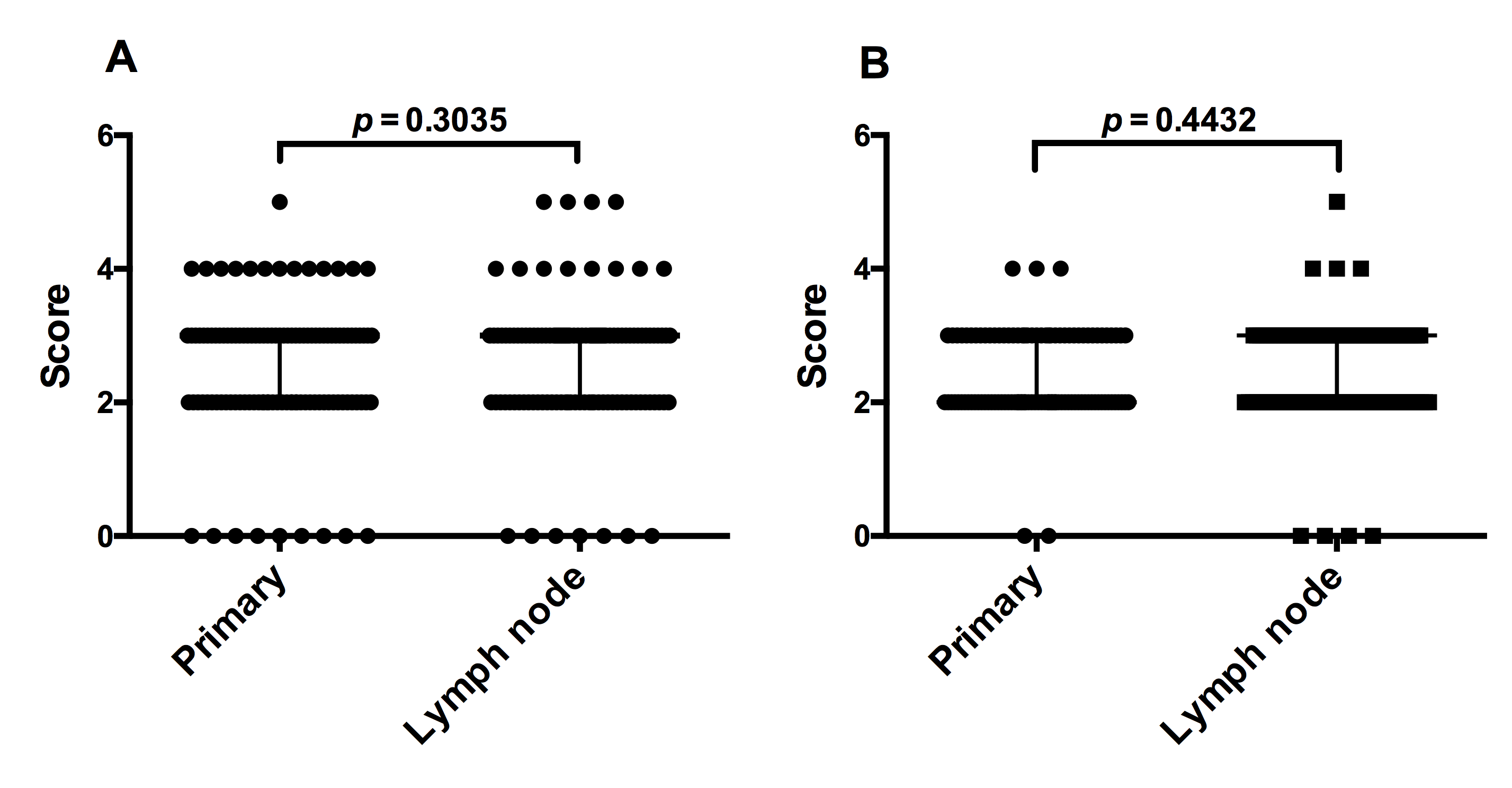

Supplement: Supplementary file 5 — Fig. S3 A Association of the immunohistochemical score of tenascin-C between the primary tumor and metastatic sites. B Association of the immunohistochemical score of OR11H1 between the primary tumor and metastatic sites. (TIFF 180 kb) [file 10434_2022_12574_MOESM5_ESM.tiff]
